# Increasing the Vase Life of Chrysanthemum Cut Flowers by Using Silver and Zinc Nanoparticles

**DOI:** 10.1155/2023/8871491

**Published:** 2023-11-30

**Authors:** Sulusi Prabawati, Noveria Sjafrina, Anna Sulistyaningrum, Eka Rahayu, Siti Mariana Widayanti, Waryat, Noor Roufiq Ahmadi, Fitri Rachmawati, Abdullah Bin Arif

**Affiliations:** ^1^Research Center for Agroindustry, National Research and Innovation Agency, Central Jakarta, Indonesia; ^2^Research Center for Food and Processing Technology, National Research and Innovation Agency, Central Jakarta, Indonesia; ^3^Indonesian Vegetable Research Institute, Ministry of Agriculture, South Jakarta, Indonesia; ^4^Research Center for Horticulture and Plantation, National Research and Innovation Agency, Central Jakarta, Indonesia

## Abstract

Cut flowers are horticultural products that have great potential to be developed. Efforts to maintain quality and extend the shelf life of cut flowers are very important to obtain a product that is accepted in the market. The main problems of chrysanthemum cut flowers are the leaves easily turning yellow, wilting, and failure to fully open flowers. This study aimed to obtain the best pulsing solution formulation that increases vase life and maintains the freshness of chrysanthemum cut flowers. Pulsing solution treatment was carried out on chrysanthemum cut flowers during the evaluation period. Pulsing solution treatment consisted of control, AgNO_3_, nano-Ag (NAg), ZnO, and nano-Zn (NZn). The results showed that NAg20 treatment increased the vase life of chrysanthemum cut flowers up to 23 days, which was 19 days longer than the control. Nano-Ag inhibits bacterial growth, flower wilting, color degradation, and carotenoids. In addition, nano-Ag increased the size of the bloom-flower diameter. Considering the results of all postharvest quality parameters mentioned above, NAg20 extends the vase life of chrysanthemum cut flowers.

## 1. Introduction

Cut flowers are horticultural products that have great potential to be developed. Chrysanthemum (*Chrysanthemum morifolium*) is one of the leading cut flowers in the international market which is widely used as decoration which belongs to the Asteraceae family [1, 2]. In addition, many consumers like chrysanthemums because of their varied shapes, colors, and sizes [1]. Efforts to maintain quality and extend the shelf life of cut flowers are very important to obtain a product that is accepted in the market [3, 4].

The vase life is an important parameter for evaluating the quality of cut flowers [5, 6] for both domestic and export markets. This technique of extending the vase life will be a great asset to both the farmer and the consumer [7]. The difference in vase life among various chrysanthemum species and cultivars is one of the most valuable characteristics determining its quality, consumer preferences, and commercial value [8]. The results showed that good postharvest application resulted in the freshness of chrysanthemum cut flowers being maintained for up to 21 days [9, 10], whereas poor postharvest handling resulted in the freshness of cut chrysanthemum flowers, only lasts up to 4–7 days [11, 12].

The main problems of chrysanthemum cut flowers are the leaves easily turning yellow, wilting, and failure of the flowers to fully open [13–15]. One of the postharvest technologies to overcome this problem is applying a pulsing solution. Pulsing solutions enriched with AgNO_3_ or ZnO have been shown to increase vase life and maintain cut flower quality [16–18]. The formulation of Ag particles as a pulsing solution can extend vase life, expand flower openings, and restore stem and flower size or petal color by balancing osmotic regulation [19]. Zinc (Zn) is an activator of certain enzymes that can regulate the antioxidant activity and increase the life of cut flower vases [20]. However, using these materials in a pulsing solution is more optimal for increasing vase life and maintaining the quality of potted flowers if the particle size of the material is nanosized [18].

Nanoparticles are expected to reduce the concentration of AgNO_3_ or ZnO in the pulsing solution. The application of nano AgNO_3_ can improve relative water absorption, reduce microbial density at the tip of the stem and delay stem clogging, reduce electrolyte leakage, and suppress malondialdehyde (MDA), superoxide dismutase (SOD), and peroxide activity (POD) [21]. The accumulation of Zn nanoparticles on the cell surface can cause bacterial cell death, where the interaction between bacterial cells and Zn nanoparticles causes electrostatic pressure on the surface of the cell membrane and breaks the flow/transport of electrons across the membrane [22]. However, research on applying nanoparticles in pulsing solutions to increase vase life and maintain the freshness of chrysanthemum cut flowers cultivated in the tropics still needs to be completed. Therefore, this study aims to obtain the best pulsing solution formulation that increases vase life and maintains the freshness of chrysanthemum cut flowers.

## 2. Materials and Methods

### 2.1. Plant Material

In this study, the yellow spray chrysanthemum of the Zembla Sunny variety was harvested in the morning at 07.00 AM in the farmer's garden in Kawungluwuk village, Sukaresmi, Cianjur district, West Java province. The criteria for harvesting flowers are 60% blooms, then sorting, and selecting those that are not defective, and the length of the stalks is uniform. The flower stalks were cut obliquely.

### 2.2. Coating Material Preparation

An aqueous solution of 0.04 M AgNO_3_ (Sigma-Aldrich) and an aqueous solution of 0.02 M ZnO (Sigma-Aldrich) were prepared. A pulsing solution was made from the starter solution following the procedure of Rahayu et al. [23]. The pulsing solution was made at 5, 10, and 20 ppm for NZn and NAg following Carrillo-López et al.'s [9] formula with modifications, and the pulsing solution was also added with commercial concentrations of sucrose 50 grams/liter and citric acid 200 ppm (Table 1). Sucrose as a source of carbohydrates and energy and organic acids were used to decrease the pH of the pulsing solution. Pulsing solutions with a low pH inhibited the growth of microbes so that they accelerated water and nutrients absorbed and maintained the freshness of cut flowers [6]. The pulsing solution was prepared according to the treatment (200 ml/glass).

**Figure 1:**
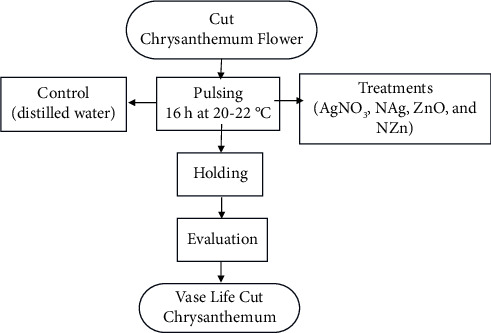
Scheme of study stages.

The flowers were then pulsed by immersing the part of the flower stalk that had been cut into the solution, with the condition that 4 cm of the stalk was submerged in the pulsing solution. Pulsing was carried out for 16 hours in a room with a temperature of 20–22°C. When pulsing was complete, the cut flowers were transferred to another container filled with distilled water for display and placed in a room with an average temperature of 20–22°C. The scheme of study stages is presented in Figure 1. Each treatment was replicated three times in a randomized complete design. Observations were made during the display for each treatment on the absorbed pulsating solution (APS), water uptake, total bacteria, petal color, flower diameters, total carotenoid, and vase life.

### 2.3. Measurements of Absorbed Pulsating Solution (APS) and Vase Life

The APS was measured using the method proposed by Veluru et al. [24]. The flower stems were placed in a 250 ml conical flask containing a 200 ml pulsing solution. The APS of the flower pieces was calculated by finding the difference in the amount of the pulsing solution before and after 16 hours. The APS was measured using a graduated glass cylinder. Finally, the APS was expressed in ml:

(1) $$\begin{array}{c} \text{APS}=Vf-Vi. \end{array}$$

*Vf* = the final volume of the solution after absorption and *Vi* = the initial volume of solution added.

Wilting of flower petals was used as a measure for determining the vase life of chrysanthemum cut flowers. The average vase life was assessed to be terminated when 80% of flowers had senesced, which was characterized by the loss of turgor followed by petal wilting [25]. The vase life was calculated by counting the days from applying the treatment (first day) until flower wilting [10]. Then, the vase life was expressed as the day.

### 2.4. Measurements of Water Uptake and Total Bacterial Count

The volume of the vases containing water without cut flowers was recorded every day during the vase life evaluation period [10]. Total days of the water uptake rate were calculated at the end of the vase evaluation period of each treatment. Finally, the water uptakes were expressed in ml.

The total bacteria were measured according to the method described by Carrillo-López et al. [9] with slight modifications. At the end of vase life, an aliquot of 1 ml of the vase solution was taken and placed in a Petrifilm Aerobic Count plate. The results are expressed in CFU/ml.

### 2.5. Measurements of the Wilted Leaves and Flower Diameter

The wilted leaves were calculated at the end of the vase life and expressed as percentages. Flower diameter was defined as the maximum width of each flower before and after blooming [25] and was measured with a ruler every day.

### 2.6. Measurements of Flower Color and Total Carotenoid

The color changes were measured according to the method described by Arif et al. [26, 27] with slight modifications. The color measurement was carried out using CR-400 Chroma Meter (Minolta Japan) and expressed lightness (*L*) and hue values on flowers. Then, the average was determined.

Total carotenoid was determined by the method of Braniša et al. [28]. The extract preparation procedure is identical to the method described by Braniša et al. [28]. A mixture of acetone water (4 : 1) was used as a solvent. The maximum absorbance was read at 470 nm.

### 2.7. Statistical Analysis

The data were analyzed using the analysis of variance. The effect of treatment on parameters was evaluated with the Duncan multiple range test (DMRT) to identify significant differences at a significance level of 5%. All statistical analyses were carried out using SAS Portable 9.13 software.

## 3. Result and Discussion

### 3.1. The APS and Vase Life

The APS was not significantly different between treatments, ranging from 32.67 ml to 36.67 ml (Table 2). This shows that the flower conditions used for the study were relatively uniform and the APS for 16 hours went well. Kader [29] reported that the absorbed pulsating solution for 24 hours in cut roses did not differ between the AgNO_3_ and NAg treatments. The APS will affect the vase life of the cut flowers.

The vase life is an important parameter for evaluating the quality of cut flowers. The NAg20 treatment showed the highest vase life of 23 days, 19 days longer than that of the control (Table 2). Silver nanoparticles have been shown to inhibit ethylene production and increase postharvest longevity of the “First Red” rose cultivar [30, 31]. Other studies have also shown that silver nanoparticles significantly inhibit xylem blockage and reduce ethylene production by suppressing the level of transcripts of the ACS1 and ACO1 ethylene biosynthetic genes, thereby increasing the vase life of *Dianthus caryophyllus* [32–34]. However, selecting of the optimum nanosilver concentrations for a particular genotype is essential to avoid phytotoxicity, which negatively impacts the vase life of cut flowers [35]. In addition, in this study, the vase life of chrysanthemum cut flowers in the NAg20 treatment was four days longer than that in the Ag25 treatment. The nanosilver treatment was more effective than Ag^+^ in the vase life of cut carnation flower, where the accumulation of silver content was higher at the tips of the stems in the nanosilver treatment, which might be due to differences in physical and chemical characteristics and/or concentration [36]. Moreover, silver and nanosilver suppressed the expression of ACS and ACO, interacted with ethylene receptors, and modulated the ethylene response, so it increased the vase life of carnations [33]. Thus, the NAg20 treatment can extend vase life up to 23 days in chrysanthemum cut flowers.

The vase life of chrysanthemum cut flowers in the nanozinc (NZn) treatment was not different from that in the control, which was 4 days (Table 1). This is inconsistent with research reports showing that zinc treatment can increase vase life in *Gerbera jamesonii* flowers [16] and lisianthus [37]. In this study, the nanozinc synthesis process did not use capping/doping materials, so resulting nanozinc became ineffective and unstable. Nanozinc synthesized using a capping/doping agent (starch) increased the shelf life of cut flowers up to three times compared to controls whose charge was unstable and experienced agglomeration to a certain extent due to lack of surface passivation [16].

### 3.2. The Water Uptake and Total Bacteria

In this study, water absorption by chrysanthemum cut stems ranged from 5 to 7 ml per day, except for the control (Table 3). The NAg20 treatment showed a stable water absorption of 147.89 ml for up to 23 days of the display period (Table 3). In this study, nanosilver particles kill bacteria that clog xylem vessels and maintain water balance. Nanosilver particles inhibit bacterial growth, enhance antioxidant activity, and promote the expression of the intrinsic plasma membrane protein genes PlPIP1: 2 and PlPIP2: 1, which help maintain water balance in *Paeonia lactiflora* cut flowers [38]. Thus, the NAg20 treatment indicated that it could maintain water balance in chrysanthemum cut flowers.

Water uptake is critical in improving cut flowers' vase life and quality [33]. Water uptake is crucial to increase vase life and cut flower quality because it is needed to maintain water balance in the stem and for flower bud opening in cut flowers [36]. Xylem is mainly responsible for transporting water to flower buds and flowers so that water absorption by cut flowers is inhibited by the proliferation and growth of microorganisms and the deposition of microbial residues in the lumen of xylem vessels [33, 35]. In addition to water uptake, total bacteria affect the vase life of cut flowers. In this study, total bacteria increased at the end of the display period (Table 4). Generally, the AgNO_3_ and NAg treatments showed that the average of total bacteria per day was lower than that of the control and Zn treatments (Table 4). The total bacteria in the NAg20 treatment on day 23 of the evaluation period were 6050 CFU/ml, which means 263 CFU/ml per day, where the total bacteria were the lowest compared to those in the other treatments (Table 4). Nanosilver particles have been shown to have stronger bacterial growth inhibition properties than the micrometric structure or oxidation state components of silver [39] due to their high surface area to volume ratio and large external area effect [40, 41]. Silver ions released by nanosilver particles interact with cytoplasmic components and nucleic acids to inhibit respiratory chain enzymes and disrupt membrane permeability, which can cause death in microorganisms [35]. In addition, the nanosilver particles kill bacteria growing in the vase solution and enter the cut flower stems through the vascular tissue and inhibit bacterial colonization at the cut ends. The proliferation of bacterial growth in xylem vessels is the leading cause of water transportation to cut flowers, so bacterial growth in xylem vessels can inhibit water uptake in vases [18, 35, 42]. Microbial growth at the tips of the stems is the main cause of clogging of xylem vessels, which prevents water absorption and consequently reduces the vase life of cut flowers [43]. Therefore, the NAg20 treatment was effective in inhibiting bacterial growth in chrysanthemum cut flowers.

### 3.3. Number of Wilted Leaves and Flower Diameter

**Figure 2:**
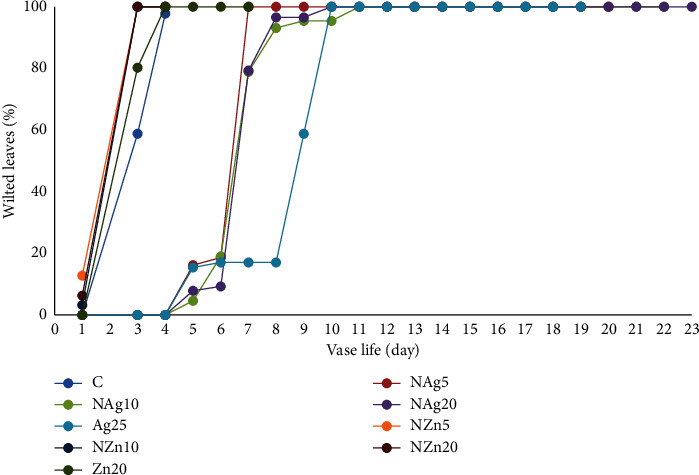
Wilted leaves in chrysanthemum during vase life.

**Figure 3:**
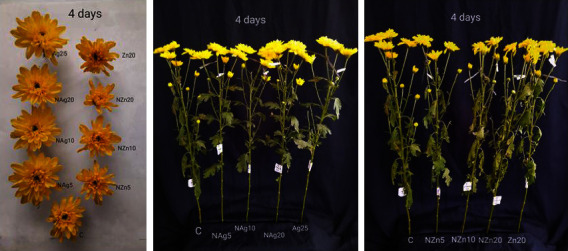
The appearance of the flower buds in Ag- and Zn-pulsing treatments and the front view of the entire flower stalk on day 4 during the evaluation period.

The freshness of cut flower leaves is an important parameter that can affect the level of cut flower consumer preference. On the other hand, leaves on flower stalks treated with NAg or AgNO_3_ began to wilt on the 5th day of the display period, and all wilted on day 7 to day 9 of the display period (Figures 2 and 3). Treatment with nanosilver particles reduced chlorophyll pigment degradation and lipid peroxidation (malondialdehyde) and increased SOD (superoxide dismutase) activity to prevent oxidative stress in *Alstroemeria* cut flower leaves [44]. In addition, the treatment of nanosilver particles also improved the postharvest quality of cut flowers by reducing weight loss and increasing the greenness index of cut leaves [45]. This shows that the nanosilver treatment inhibits leaf wilting in cut flowers.

**Figure 4:**
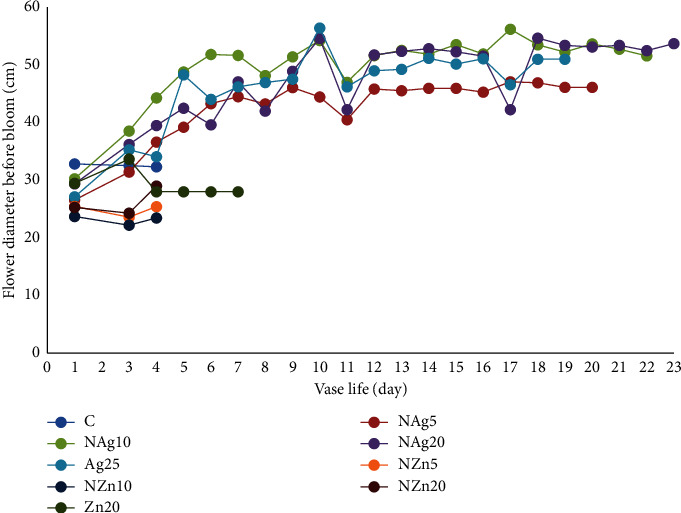
Changes in the diameter of blooming flowers during vase life.

**Figure 5:**
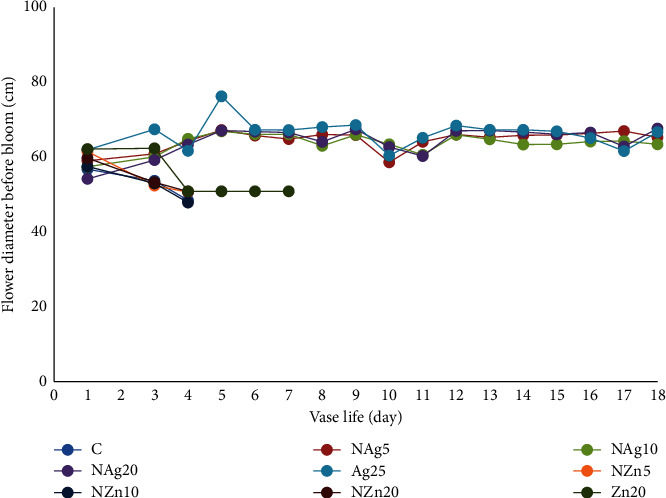
Changes in the diameter of flowers that are not in full bloom during vase life.

In one sprig of spray-type chrysanthemum, there are several flower buds, namely, full bloom, half bloom, and buds. At the start of the display period, the average flower diameter at full bloom was 52–67 mm. At 4 days of the display period, the flowers in the control, ZnO, and NZn treatments showed wilted flowers, drooping petals, and decreased flower diameter (Figure 4). This is presumably due to the occurrence of barriers to water absorption and translocation of food reserves, especially carbohydrates, to the floral organs. Furthermore, pulsing treatment of AgNO_3_ and NAg at various concentrations showed flower diameters of 63–67 mm up to 18–23 days. In the quarter-to-half blooms, the flower diameter was 23–30 mm and the AgNO_3_ and NAg treatments showed an increase in a flower diameter of 34–44 mm (Figure 5). Flower diameter in the AgNO_3_ and NAg treatments continued to increase until full bloom, where the flower diameter reached 51–56 mm. However, the diameter of the flower was smaller than the flower that was in full bloom from the start. Nanosilver particles kill bacteria that clog xylem vessels and support greater water uptake so that they can translocate water and carbohydrates to flowers [9]. Leaves that quickly turn yellow and wither can cause smaller flower diameters, which eventually cause the flowers to wilt. The promotion of flower opening, as evidenced by the yellowing and wilting of leaves, indicates increased transport of dissolved carbohydrates and sugars from leaves to flowers [9]. Therefore, the NAg treatment increased flower size and delayed flower wilting.

### 3.4. Flower Color and Total Carotenoid

**Figure 6:**
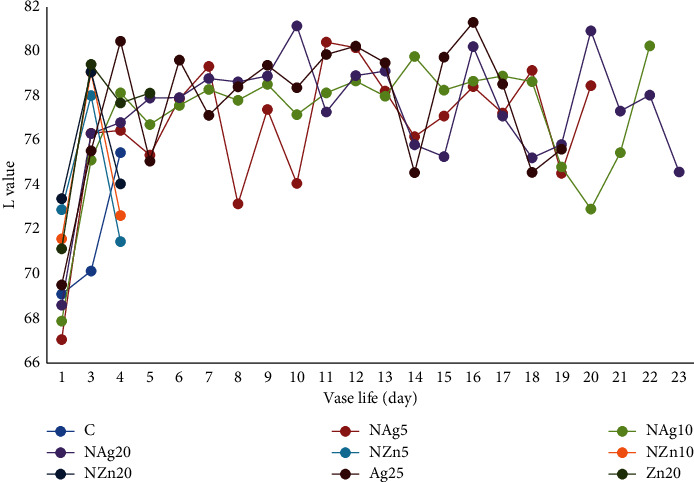
Changes in lightness *L* color of flowers during vase life.

**Figure 7:**
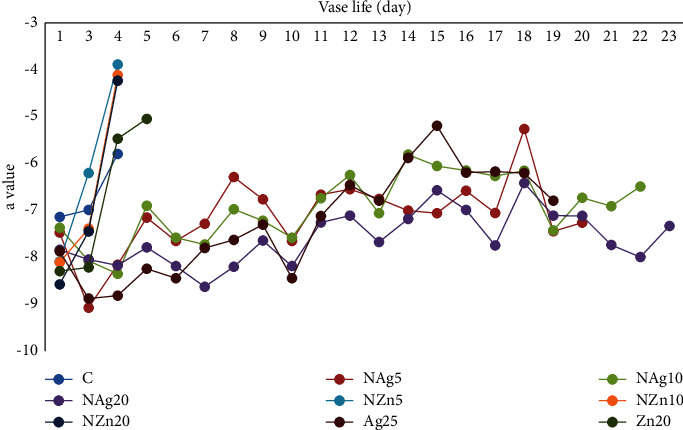
Changes in *a*-value color of flowers during vase life.

**Figure 8:**
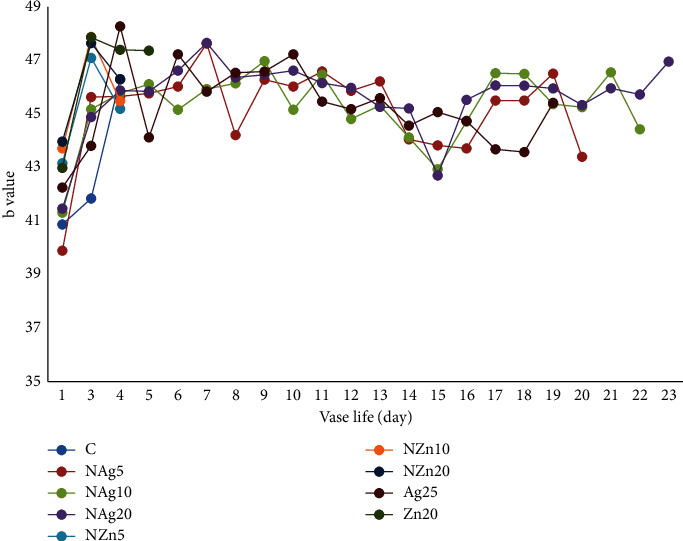
Changes in *b*-value color of flowers during vase life.

Color is an important visual parameter influencing consumer acceptance of horticultural commodities [26, 27]. The color of the petals on the Zembla Sunny spray chrysanthemum is yellow. Changes in the color of flower petals in the NAg20 treatment tended to show no significant changes during vase life (Figures 6–8). It is suspected that the NAg treatment affects the synthesis and production of ethylene. Naing et al. [33] reported that silver and nanosilver increased the vase life of carnations because they suppressed the expression of ACS and ACO, interacted with ethylene receptors, and modulated the ethylene response. Ethylene is a hormone that directly affects pigmentation and color degradation [26, 27]. Therefore, in this study, the lightness of the color of the flower petals in the NAg20 treatment was paler than that in the other treatments.

In addition, this study also observed the carotenoid content in chrysanthemum cut flowers during vase life. Carotenoids are pigments that are yellow, orange, or red. The carotenoid content increased in control, ZnO, AgNO_3_, and NZn (Table 5). In contrast, the content of carotenoids in the NAg20 treatment tended to decrease (Table 5). This indicates that the process of carotenoid degradation in the NAg treatment is inhibited. The inhibition of the color degradation process is caused by the ethylene synthesis process inhibited but increases the shelf life of most horticultural commodities [26, 27].

## 4. Conclusion

The pulsing solution significantly affected postharvest physiological changes during vase life in chrysanthemum cut flowers. The NAg20 treatment increased the vase life of the chrysanthemum cut flowers up to 23 days, which was 19 days longer than that in the control. NAg inhibits bacterial growth, flower wilting, color degradation, and carotenoids. In addition, nano-Ag increased the size of the bloom-flower diameter. These data are very important because they show the complexity in determining the appropriate pulsing solution treatment to extend the vase life of the chrysanthemum cut flower. This research can be used as a material for consideration for cut chrysanthemum industry players to determine the ideal pulsing solution treatment for the chrysanthemum cut flowers, which can meet consumer preferences and balance the interests of farmers.

## Figures and Tables

**Table 1:** The formula composition of the pulsing solution.

Code	Formula composition of pulsing solution
Control (C)	Distilled water
NAg5	5 mg nano-Ag + citric acid + sucrose
NAg10	10 mg nano-Ag + citric acid + sucrose
NAg20	20 mg nano-Ag + citric acid + sucrose
NZn5	5 mg nano-Zn + citric acid + sucrose
NZn10	10 mg nano-Zn + citric acid + sucrose
NZn20	20 mg nano-Zn + citric acid + sucrose
Ag25	25 mg AgNO_3_ + citric acid + sucrose
Zn20	20 mg ZnO + citric acid + sucrose

**Table 2:** The APS and vase life of chrysanthemum cut flowers.

Treatment	APS (ml/16 h)	Vase life (day)
C	22.00^a^	4.00^d^
NAg5	20.67^a^	20.22^ab^
NAg10	19.60^a^	22.00^ab^
NAg20	21.27^a^	23.00^a^
Ag25	20.33^a^	19.33^b^
NZn5	21.20^a^	4.00^d^
NZn10	20.07^a^	4.00^d^
NZn20	21.07^a^	4.00^d^
Zn20	20.93^a^	7.00^c^

*Notes*. The numbers followed by the same letter in the same column indicate no significant difference by the Duncan extension multiple range test (*P* < 0.05).

**Table 3:** Water uptake in chrysanthemum cut flowers.

Treatment	Water uptake (ml)						Average per days
	Day 4	Day 7	Day 19	Day 20	Day 22	Day 23	
C	47.78	—	—	—	—	—	11.95^a^
NAg5	—	—	—	125.78	—	—	6.29^c^
NAg10	—	—	—	—	145.33	—	6.61^c^
NAg20	—	—	—	—	—	147.89	6.43^c^
Ag25	—	—	146.00	—	—	—	7.68^b^
NZn5	24.00	—	—	—	—	—	6.00^c^
NZn10	21.33	—	—	—	—	—	5.33^d^
NZn20	22.11	—	—	—	—	—	5.53^d^
Zn20	—	33.33	—	—	—	—	4.76^d^

*Notes*. The numbers followed by the same letter in the same column indicate no significant difference by the Duncan extension multiple range test (*P* < 0.05).

**Table 4:** Total bacteria in chrysanthemum cut flowers.

Treatment	Total bacteria (CFU/ml)						Average per days
	Day 4	Day 7	Day 19	Day 20	Day 22	Day 23	
C	455	—	—	—	—	—	113^c^
NAg5	—	—	—	5200	—	—	260^c^
NAg10	—	—	—	—	15500	—	704^bc^
NAg20	—	—	—	—	—	6050	263^c^
Ag25	—	—	5550	—	—	—	292^c^
NZn5	7300	—	—	—	—	—	1820^ab^
NZn10	10800	—	—	—	—	—	2730^a^
NZn20	5500	—	—	—	—	—	1370^abc^
Zn20	—	6250	—	—	—	—	892^bc^

*Notes*. The numbers followed by the same letter in the same column indicate no significant difference by the Duncan extension multiple range test (*P* < 0.05).

**Table 5:** Total carotenoids in chrysanthemum cut flowers.

Treatment	Total carotenoids (ppm)							Distinction
	Day 0	Day 4	Day 7	Day 19	Day 20	Day 22	Day 23	
C	88.35	127.05	—	—	—	—	—	38.70
NAg5	88.35	—	—	—	78.14	—	—	−10.21
NAg10	88.35	—	—	—	—	88.91	—	0.56
NAg20	88.35	—	—	—	—	—	82.44	−5.91
Ag25	88.35	—	—	111.39	—	—	—	23.04
NZn5	88.35	126.12	—	—	—	—	—	37.77
NZn10	88.35	186.33	—	—	—	—	—	97.98
NZn20	88.35	154.89	—	—	—	—	—	66.54
Zn20	88.35	—	126.75	—	—	—	—	38.40

## Data Availability

The data can be found in the National Research and Innovation Agency, Indonesia (https://www.brin.go.id).
